# Perception, signaling and molecular basis of oviposition-mediated plant responses

**DOI:** 10.1007/s00425-013-1908-y

**Published:** 2013-06-08

**Authors:** Philippe Reymond

**Affiliations:** Department of Plant Molecular Biology, University of Lausanne, 1015 Lausanne, Switzerland

**Keywords:** Insect oviposition, Hypersensitive response, Herbivory, Egg elicitors, SA pathway, JA pathway, Defense gene expression, Plant volatiles

## Abstract

Eggs deposited on plants by herbivorous insects represent a threat as they develop into feeding larvae. Plants are not a passive substrate and have evolved sophisticated mechanisms to detect eggs and induce direct and indirect defenses. Recent years have seen exciting development in molecular aspects of egg-induced responses. Some egg-associated elicitors have been identified, and signaling pathways and egg-induced expression profiles are being uncovered. Depending on the mode of oviposition, both the jasmonic acid and salicylic acid pathways seem to play a role in the induction of defense responses. An emerging concept is that eggs are recognized like microbial pathogens and innate immune responses are triggered. In addition, some eggs contain elicitors that induce highly specific defenses in plants. Examples of egg-induced suppression of defense or, on the contrary, egg-induced resistance highlight the complexity of plant–egg interactions in an on-going arms race between herbivores and their hosts. A major challenge is to identify plant receptors for egg-associated elicitors, to assess the specificity of these elicitors and to identify molecular components that underlie various responses to oviposition.

## Introduction

Insect eggs display a variety of forms, decorations and colors. In addition, they contain a wide range of defensive chemicals that allow them to survive in the most fragile stage of an insect’s life. Being immobile, eggs are indeed vulnerable to predators and bacterial infections and it is crucial that they go through completion of the embryo’s development without any harm. Although some herbivorous insects lay their eggs on soil, most insects lay eggs on plant parts (leaves, petioles, stems, tree barks), relying on specific plant chemicals that allow females to carefully choose the appropriate host (Schoonhoven et al. 2005). Oviposition process can vary among insects, ranging from loose or tight attachment to the leaf surface, insertion in cavities after scratching the leaf cuticle or deposition after the mesophyll tissue is wounded. In most cases, eggs are glued or covered by secretions derived from accessory glands or the oviduct (Hilker et al. 2002b). A careful examination of the egg–leaf interface indicated that secretions are either in close contact with the cuticle, penetrate in the leaf through stomata or are directly in contact with mesophyll cells (Müller and Rosenberger 2006). For years, plants were only considered as an inert substrate for oviposition but several studies demonstrated that, upon recognition of egg-derived specific elicitors, plants trigger direct defenses or indirect defenses. Briefly, direct defenses consist notably of necrosis on oviposited leaves that restricts egg attachment, hatching or development (Shapiro and DeVay 1987; Balbyshev and Lorenzen 1997), tumor-like structures called “neoplasm” derived from cell division of undifferentiated tissue that lifts the eggs and presumably reduces egg or larval survival (Doss et al. 2000; Cooper et al. 2005; Petzold-Maxwell et al. 2011), the massive growth of wound tissue that crushes beetle eggs (Desurmont et al. 2011), the production of toxic molecules like benzyl benzoate (Seino et al. 1996; Yamasaki et al. 2003) and iridoid glycoside (Peñuelas et al. 2006). Indirect defenses include the emission of a bouquet of volatile compounds that attract egg parasitoids. Studies in elm and pine demonstrated that specific egg-induced terpenoids are attractive to parasitoids, both in the laboratory and in the field (Hilker et al. 2002a; Mumm et al. 2003; Mumm and Hilker 2005; Büchel et al. 2011; Wegener et al. 2001). Eggs also induce indirect defenses by triggering changes in leaf surface chemistry as in Brassicaceae. Parasitoids are arrested in the vicinity of eggs and spend more time searching for their host than on non-oviposited leaves (Fatouros et al. 2005, 2007, 2009). Finally, in certain insect species, wounding of the tissues by ovipositing females, addition of oviduct secretions and the release of egg components lead to the formation of galls, which consist of atypical plant tissue structures that provide shelter and food to hatching larvae (Hilker et al. 2002b; Stone and Schönrogge 2003). For a comprehensive information of oviposition-induced physiological, morphological and developmental changes in plants and their effects on interactions with the second and third trophic level, readers are referred to excellent reviews by Hilker et al. (2002b), Hilker and Meiners (2006), Fatouros et al. (2008b) and Hilker and Meiners (2010, 2011). In this review, a recent progress in the understanding of how plants perceive insect eggs deposited on leaves and mount a defense response, with a particular emphasis on molecular events underlying these processes, is addressed.

## Perception of egg-derived compounds

**Fig. 1:**
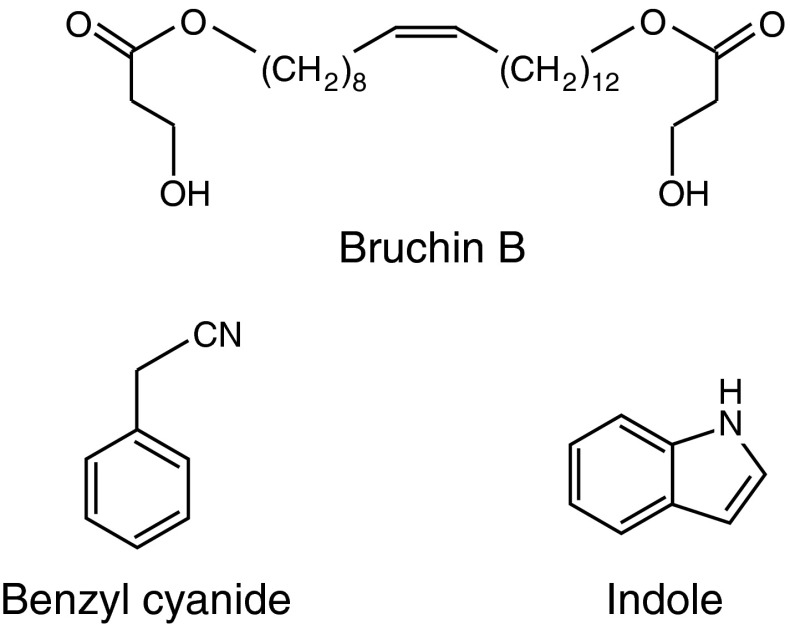
Structures of known egg-associated elicitors. Bruchins are found in oviposition fluids of pea weevils. They are C_22_–C_24_ long-chain α,ω-diols, esterified at one or both ends with 3-hydroxypropanoic acid and induce neoplasm formation when applied to pea pods. Benzyl cyanide is a male-derived anti-aphrodisiac molecule found in accessory gland secretions coating eggs of *Pieris brassicae*. It induces the arrest of the egg parasitoid *Trichogramma brassicae* on Brassicaceae plants. Indole is another anti-aphrodisiac molecule found in *Pieris* *rapae* eggs that also arrests *T.* *brassicae*

During feeding, insect larvae release compounds from oral secretions in the wound site that induce direct and indirect defenses. These so-called “elicitors” have different chemical structures and, in some cases, consist of plant components that are modified in the insect midgut (Mithoefer and Boland 2008; Wu and Baldwin 2010). Concerning egg-derived elicitors, much less is known about their chemical nature. The first isolation and characterization of an egg elicitor was from adult bruchid weevils. Active molecules, referred to as “bruchins”, are C_22_–C_24_ long-chain α,ω-diols esterified at one or both ends with 3-hydroxypropanoic acid (Fig. 1). Bruchins were shown to induce neoplasms in legumes and were only found in bruchid species, illustrating a quite narrow specificity (Doss et al. 2000). In addition, egg extracts and oviposition fluids stimulated neoplasm formation (Doss et al. 1995). The function of bruchins in insect physiology or development is not known but, considering that plants have evolved mechanisms to recognize mainly non-self molecules that are generally indispensable for the attacker, these elicitors may play a crucial role that deserves further investigation.

The elicitor responsible for surface chemical changes by *Pieris* *brassicae* eggs on *Brassica oleracea* var *gemmifera* was identified in accessory reproductive gland (ARG) secretions released with eggs by female butterflies. This molecule is benzyl cyanide (BC, Fig. 1), a male-derived anti-aphrodisiac. BC mimics the egg-induced arrest of *Trichogramma brassicae* parasitoid wasp when applied to *B.* *oleracea* and *Arabidopsis* leaves (Fatouros et al. 2008a; Blenn et al. 2012). In addition, BC was shown to be a cue by which *T.* *brassicae* wasps locate *P.* *brassica*e butterflies to be transported to the oviposition site (Fatouros et al. 2005). Methyl salicylate and indole are other anti-aphrodisiac substances that are transferred to ARG of *Pieris* *rapae* female butterflies. Interestingly, indole (Fig. 1) was found only in ARG extracts from mated female and was able to arrest *T.* *brassicae* when applied to *B.* *oleracea* (Fatouros et al. 2009). Anti-aphrodisiacs enhance reproductive success of males and prevent harassment of mated females during oviposition. Although it makes sense from an ecological point of view, it is again striking at the physiological level that such essential molecules are recognized by plants for their own defense.

In the case of volatile emission after oviposition by the pine sawfly *Diprion* *pini*, the elicitor was found in oviduct secretions coating the eggs when females insert them into a slit in the pine needle. This elicitor is a protein or a peptide, or an associated compound, since the activity was lost after treatment with proteinase K (Hilker et al. 2005). Oviduct secretions covering eggs from the elm leaf beetle *Xanthogaleruca* *luteola* were also shown to contain an elicitor that triggers volatile emission when applied to an artificially scratched elm leaf surface (Meiners and Hilker 2000). This elicitor is also of proteinaceous nature (discussed in Hilker and Meiners 2010). Finally, emission of volatiles in maize landrace varieties after oviposition by *Chilo partellus* could be mimicked by applying an ethanolic extract of the adhesive substance underneath the eggs (Tamiru et al. 2011).

Up to now, the small numbers of known egg-derived elicitors are all associated with secretions that are released with the eggs. On the contrary, an elicitor from *P.* *brassicae* eggs that induce defense responses in *Arabidopsis* appears to be contained in the egg (Little et al. 2007). Using an *Arabidopsis* transgenic line containing the promoter of the egg-induced gene *PR1* coupled to the β-glucuronidase (GUS) reporter gene, it was shown that application of soluble *P. brassicae* egg extracts activated the reporter gene and that this effect was resistant to boiling (Little et al. 2007). The GUS reporter line also responded to application of egg extracts from distantly related insects, including *Spodoptera* *littoralis*, *Drosophila* *melanogaster* (Bruessow et al. 2010), and *X.* *luteola* (F. Bruessow, unpublished). Empty *P.* *brassicae* eggshells were not active (Bruessow et al. 2010), nor were compounds left on the plant surface after eggs had been quickly removed after oviposition (Bruessow, unpublished), suggesting that gene-induction activity resides within the egg. The elicitor is resistant to proteinase K treatment and is enriched in egg lipids (Bruessow et al. 2010). Initial purification of total lipids with solid phase extraction showed that a fraction eluting with 100 % MeOH strongly activated the GUS reporter gene and enhanced the expression of egg-responsive genes (Gouhier-Darimont et al. 2013). This relatively simple assay should allow in the future to purify the elicitor to homogeneity and define its chemical structure. In contrast to egg responses to specific elicitors from insects that are associated with a relatively small number of plant species (Hilker and Meiners 2010), the observation that egg extracts from distantly related insect species, specialists or generalists activate the same reporter gene is an indication that some generic egg molecules are recognized by the plant. Intriguingly, this is analogous to the detection of pathogen-associated molecular patterns (PAMPs) from bacterial and fungal pathogens that activate a basal defense called pattern-triggered immunity (PTI) (Boller and Felix 2009) and suggest that plants respond similarly to insect eggs and microbes at the molecular level. Clearly, more work will be needed to enlarge the repertoire of chemically defined egg elicitors and to assess their respective specificity.

Although it is generally assumed that plants detect elicitors through cell-surface receptors, no such protein has been identified yet, neither for elicitors from insect oral secretions nor for egg elicitors. An initial attempt to identify a plant receptor for the *P.* *brassicae* lipid-derived elicitor was carried out. Based on the assumption that this receptor belongs to the class of receptor-like kinases (RLK), which are known plasma membrane-located receptors for PAMPs (Dardick and Ronald 2006), T-DNA insertion lines for 41 egg-induced RLKs from *Arabidopsis* (Little et al. 2007) were screened for their responsiveness toward egg extract application. One line mutated in a gene encoding LecRK-I.8, which is an l-type lectin receptor kinase, showed a strong, although not complete, reduction of *PR1* expression in response to egg extract treatment (Gouhier-Darimont et al. 2013). This result suggested that LecRK-I.8 plays a role in the perception of egg-derived elicitors in *Arabidopsis*. LecRKs are postulated to bind to carbohydrate containing ligands, but the presence of a conserved hydrophobic pocket does not exclude other more hydrophobic ligands (Barre et al. 2002). Further chemical characterization of the *P.* *brassicae* egg elicitor and a demonstration of its binding to LecRK-I.8 will be crucial to understand the early phases of egg recognition. It would also be interesting to test if LecRK-I.8 is involved in leaf surface chemical changes that are induced by *P.* *brassicae* oviposition in *Arabidopsis* (Blenn et al. 2012).

## Signaling of egg detection

Plants must rely on signaling molecules to transduce information from egg perception to gene expression and further biological responses. Known signals in plant defense are jasmonic acid (JA), salicylic acid (SA) and ethylene (ET) (Reymond and Farmer 1998). Over the past few years, the JA pathway has been shown to be crucial for plant resistance to feeding insect larvae (Howe and Jander 2004). JA treatment was shown to mimic egg-induced emission of volatiles in pine and elm that resulted in attraction of egg parasitoids (Meiners and Hilker 2000; Wegener et al. 2001; Hilker et al. 2002a). Oviposition by *X.* *luteola* in elm and bruchin treatment of pea pods induced JA biosynthesis genes (Doss 2005; Büchel et al. 2012). *H.* *zea* eggs triggered the expression of the known JA-responsive gene *PIN2* in tomato, but JA levels were not altered (Kim et al. 2012). A tomato mutant *def1* that is unable to accumulate JA in response to wounding or herbivory showed a much higher hatching rate of the phytophagous mite *Tetranychus urticae*, suggesting that the JA pathway enhanced egg mortality (Ament et al. 2004). Thus, JA signaling seems to be involved in some responses to oviposition, but more molecular and genetic data are needed to better understand the precise involvement of this pathway.

On the contrary, response to oviposition by *P.* *brassicae* on *Arabidopsis*, where eggs are only deposited on the leaf surface without wounding, appears to be controlled by a different signaling pathway. SA accumulated at high levels underneath the eggs and many SA-responsive genes were induced by oviposition (Little et al. 2007; Bruessow et al. 2010) (see below). Recently, induction of *PR1* and other SA-responsive genes by *P.* *brassicae* egg extract was shown to be controlled by EDS1 and NPR1, which are central regulators of the SA pathway (Vlot et al. 2009). This induction was abolished in the SA-deficient mutant *sid2*-*1* (Gouhier-Darimont et al. 2013). These findings demonstrate the importance of the SA pathway in response to egg-derived elicitors. In addition, given that *P.* *brassicae* eggs activate early PAMP responses and that detection of microbes activates the SA pathway (Vlot et al. 2009), there are intriguing similarities between detection of insect eggs and PTI in *Arabidopsis*.

The possible involvement of ET in egg signaling was tested in pine. Needles were wounded and treated with oviduct secretions from *D.* *pini*, a treatment that mimics oviposition. ET emission in systemic needles was significantly reduced compared to control needles (Schröder et al. 2007). The link between ET emission in oviposited plants and gene expression or defense responses was however not investigated further.

Reactive oxygen species (ROS) constitute other important signaling molecules in defense. In response to oviposition, *Arabidopsis* plants accumulate high levels of the ROS superoxide (O_2_^·−^) and hydrogen peroxide (H_2_O_2_) (Little et al. 2007; Gouhier-Darimont et al. 2013). In addition, egg-induced *PR1* expression was dependent on ROS accumulation that required EDS1 activity and the SA pathway (Gouhier-Darimont et al. 2013). In plants, ROS are mainly generated by the action of NADPH oxidases that produce O_2_^·−^ in the apoplast. Two Arabidopsis NADPH oxidases, RBOHD and RBOHF, play a key role in signaling during bacterial infections (Marino et al. 2012). Single *rbohD* and *rbohF* mutants as well as *rbohD/F* double mutant exhibited wild-type production of ROS in response to egg extract suggesting that RBOHD and RBOHF do not play a role in signaling events triggered by oviposition (Gouhier-Darimont et al. 2013). Oviposition by the fruitworm moth *Helicoverpa zea* and by the anthocorid predator *Orius* *laevigatus* elicited H_2_O_2_ accumulation underneath eggs in tomato leaves (De Puysseleyr et al. 2011; Kim et al. 2012). Although ROS accumulation is often associated to defense signaling, these compounds might also have direct antimicrobial activity (Dat et al. 2000). In addition, since *O.* *laevigatus* is also known to feed on plant tissues, egg-induced ROS accumulation might also target adults that have hatched from the eggs. Whether ROS accumulation is toxic to insect eggs is unknown and will deserve further investigation.

Interestingly, whereas responses to eggs in *Arabidopsis* and *Brassica* sp. are restricted to the oviposition site or in close vicinity, volatile emission in pine, elm and maize can be induced systemically. This systemic response can also be reproduced by JA treatment, suggesting that what distinguishes these two contrasting responses is the fact that in the latter cases oviposition is accompanied by wounding or scratching of leaf tissue. Up to now, no information is available on the nature of the systemic signal that triggers volatile emission after oviposition, but future research might unravel whether it is simply a JA-dependent systemic wound signal or whether it is more specific to the recognition of egg elicitors.

Current knowledge on signaling of egg-induced responses indicates that two antagonistic pathways, the JA and the SA pathways, play a role in transducing information about the presence of eggs on plants and that ROS are also involved. The JA pathway seems to be prominent in cases where oviposition is accompanied by wounding of the leaf, whereas the SA pathway was shown to be involved when eggs are only deposited onto the surface without any apparent damage. It is not yet known whether these two types of signaling are mutually exclusive and represent a specific plant strategy in response to different elicitors. However, the observation that, besides a majority of JA-responsive genes, oviposition by elm leaf beetles induced some SA-responsive genes, including genes encoding pathogenesis-related proteins (PR1, PR2, PR3, and PR10) (see below), suggests that the JA-dependent wound-induced response might work in parallel to an SA-dependent egg-induced response. The use of signaling mutants in plant–insect interactions where oviposition is done by damaging leaf tissue might help discriminate molecular changes that are specific to each type of stimulus.

## Egg-induced changes in gene expression

Oviposition induces various morphological, physiological and chemical responses in plants. These responses are most likely the result of changes in gene expression that have only recently started to be investigated. Differential display performed on cDNAs from pea pods treated with bruchin identified several genes that were upregulated a few hours after application. One gene encoded a cytochrome P450 belonging to the isoflavone synthase family and, accordingly, levels of the isoflavone pisatin increased after bruchin treatment (Cooper et al. 2005). Since pisatin is a known defense compound in pea, this finding suggested that bruchins trigger a chemical defense response in addition to neoplasm formation (Cooper et al. 2005). In tomato, there was a strong induction of the defense gene *PIN2*, encoding an anti-insect proteinase inhibitor (Ryan 1990), under and in the vicinity of *H. zea* eggs (Kim et al. 2012). Oviposition by the sawfly *D. pini* induced the expression of two sesquiterpene synthase genes in *Pinus sylvestris*, *PsTPS1* and *PsTPS2*, and this was correlated with the attraction of the egg parasitoid *Chrysonotomyia ruforum* (Köpke et al. 2008, 2010; Beyaert et al. 2012). However, these two proteins were shown in vitro to synthesize (E)-β-caryophyllene and α-humulene (PsTPS1), and 1(10),5-germacradiene-4-ol (PsTPS2) (Köpke et al. 2008), but not β-farnesene, which was the only terpenoid that accumulated specifically in response to *D.* *pini* eggs (Mumm et al. 2003). A β-farnesene synthase gene (*PsTPS5*) was further cloned but its expression was not altered by oviposition (Köpke et al. 2010). More work is thus necessary to understand how the regulation of sesquiterpene synthase gene expression is correlated with an attractive terpenoid bouquet in pine.

The first large-scale study of egg-induced transcriptional changes was performed with *Arabidopsis* whole-genome DNA microarrays. Expression of hundreds of genes was altered over a period of 3 days after oviposition by *P.* *brassicae* on *Arabidopsis* (Little et al. 2007). Induced genes included defense proteins, regulators of cell death and innate immunity, genes responding to biotic and abiotic stresses, and genes involved in the production of defense secondary metabolites. For example, a gene encoding a callose synthase was strongly upregulated. Accordingly, oviposition led to a strong deposition of callose, a β-(1,3)-glucan polymer, underneath the eggs (Little et al. 2007). Callose plays an important defensive role against microbial pathogens (Luna et al. 2011), but its function in response to eggs is still unknown. Repressed genes were mainly involved in cell wall metabolism, cutin biosynthesis and photosynthesis (Little et al. 2007). Interestingly, egg deposition by the sawfly *D. pini* was found to reduce the net photosynthetic activity of *P.* *sylvestris* (Schröder et al. 2005). A striking finding was that the expression profile of oviposited leaves was extremely different from the profile obtained after herbivory with *P. rapae* larvae. Oviposition-induced genes were similar to those induced during bacterial or fungal infections (Little et al. 2007). These results reinforced the hypothesis that eggs are recognized more like pathogens than like herbivores in *Arabidopsis*. Further analysis of selected genes indicated that these expression changes occur mainly underneath or in the vicinity of egg deposition (Little et al. 2007; Bruessow et al. 2010). Whole-genome analysis of gene expression in response to oviposition and egg extract treatment yielded overlapping transcript profiles (Bruessow, unpublished), supporting the idea that the observed changes are due to egg-derived elicitors. Egg extract or a purified fraction from total lipids induced PAMP-responsive genes 3 h after treatment, indicating that egg-derived elicitors activate early genes that are common to the PTI response (Gouhier-Darimont et al. 2013).

*Arabidopsis* microarrays were also used to assess expression changes in *B.* *oleracea* var *gemmifera* leaves following oviposition by *P. brassicae* or BC application. Both experiments yielded a similar transcription profile and revealed 42 induced genes and 32 repressed genes (Fatouros et al. 2008a). Genes involved in cell wall metabolism and transport were upregulated by oviposition. Recently, analysis of epicuticular wax composition revealed quantitative rather than qualitative changes. Oviposited leaves had significantly higher levels of the C_34_ fatty acid tetratriacontanoic acid whereas they had reduced amounts of the C_24_ fatty acid tetracosanoic acid when compared with untreated controls (Blenn et al. 2012). More work will be necessary to link the differentially expressed genes with the observed leaf surface chemical changes and to demonstrate that they play a role in arresting egg parasitoids.

To investigate how *X. luteola* oviposition affects elm leaf transcriptional profile and leads to attraction of egg parasitoids, a large-scale study was conducted on cDNA libraries after oviposition, feeding, manual transfer of eggs, and methyl-JA (MeJA) treatment (Büchel et al. 2012). High-throughput sequencing produced ca. 50,000 unique transcripts. Overall, oviposition reduced the expression of photosynthesis genes, similar to *Arabidopsis* response to *P. brassicae* eggs (Little et al. 2007), and induced the expression of many defense-related genes, including pathogenesis-related proteins (chitinases and glucanases) and genes involved in abiotic stress and phytohormone signaling (Büchel et al. 2012). Curiously, since *X. luteola* oviposition triggers the emission of terpenoids (Wegener et al. 2001), very few transcripts involved in terpenoid metabolism were identified. There was also a significant overlap between oviposition- and MeJA-induced profiles (Büchel et al. 2012). *X. luteola* females scratch the leaf surface before laying eggs and it is thus plausible that a significant portion of the observed expression changes was due to a wound response. Indeed, JA is a well-known signal controlling wound-responsive genes (Howe 2004) and this might explain the similarity between oviposition and MeJA profiles. To support this, a manual transfer of egg clutches to scratched leaves yielded only minor differences in gene expression compared to control plants (Büchel et al. 2012). However, the observation that only oviposition by *X. luteola*, and not leaf scratching alone, renders elm leaves attractive to parasitoids suggests that either this response does not depend on transcription or that induction of specific transcripts was not detected in this particular experiment. Although at the molecular level, it appears difficult to distinguish wounding from specific egg effects in cases where insect females insert their eggs into leaves, on the ecological level both factors occur during oviposition and provide information to the plant on the presence of the egg.

In recent years, several studies have thus convincingly shown that oviposition triggers massive transcriptional reprogramming. It is, however, still unclear how these changes correlate with direct or indirect defenses and which genes are responsible for egg-specific responses.

## Similarities between responses to eggs and microbial pathogens

Contrary to elicitors from insect oral secretions that activate mainly the JA pathway and accompanying antiherbivore defenses (Wu and Baldwin 2010), the perception of egg-derived elicitors and some aspects of downstream signaling events share intriguing similarities with plant responses to microbial pathogens. One of the frequently observed direct defense response induced by oviposition is the development of necrosis at the site of egg deposition, which can hamper egg development and hatching of larvae. Because of the analogy with the hypersensitive response (HR), a pathogen-triggered programmed cell death that restricts the growth of pathogens at the infection site (Lam et al. 2001), this reaction was originally referred to HR (Shapiro and DeVay 1987). However, in plant innate immunity, HR is the consequence of the specific recognition of pathogen effectors by plant-encoded resistance genes (Jones and Dangl 2006). During coevolution of plants and pathogens, PTI has become a target for microbial effectors that interfere with plant defense to enhance their own virulence. These effectors were in turn detected by plant resistance genes, a process called effector-triggered immunity (ETI), leading to an exacerbated defense response, culminating in HR and containment of the invader (Jones and Dangl 2006). Given that such specific molecular recognition has not yet been demonstrated in egg-induced necrosis, it would thus be more appropriate to call it an “HR-like necrosis”. However, several studies indicate that egg-induced HR-like necrosis might be under similar genetic control. Eggs of the white-backed planthopper *Sogatella* *furcifera* trigger a watery lesion accompanied by dark brownish discoloration in the Reiho rice variety but not in others (Suzuki et al. 1996). Eggs of *Heliothis subflexa*, a specialist noctuid moth that is adapted to *Physali*s sp., induce necrosis on *P.* *angulata* and *P.* *pubescens* leaves but not on non-host plants (Petzold-Maxwell et al. 2011). *Brassica nigra* plants respond differently to oviposition by the specialist *P. brassicae* and the generalist *Mamestra brassicae*. After oviposition, 50 % of plants developed HR-like necrosis under *P. brassicae* eggs and this was correlated with egg desiccation or drop-off (Fatouros et al. 2012). On the contrary, *M.* *brassicae* eggs did not induce any HR-like necrosis. Electron microscopy micrographs revealed that *P. brassicae* eggs were attached much more firmly to the leaf surface than *M.* *brassicae* eggs, providing one explanation for this difference (Fatouros et al. 2012). But an alternative hypothesis is that *P.* *brassicae* eggs contain specific effectors that trigger HR-like necrosis.

**Fig. 2:**
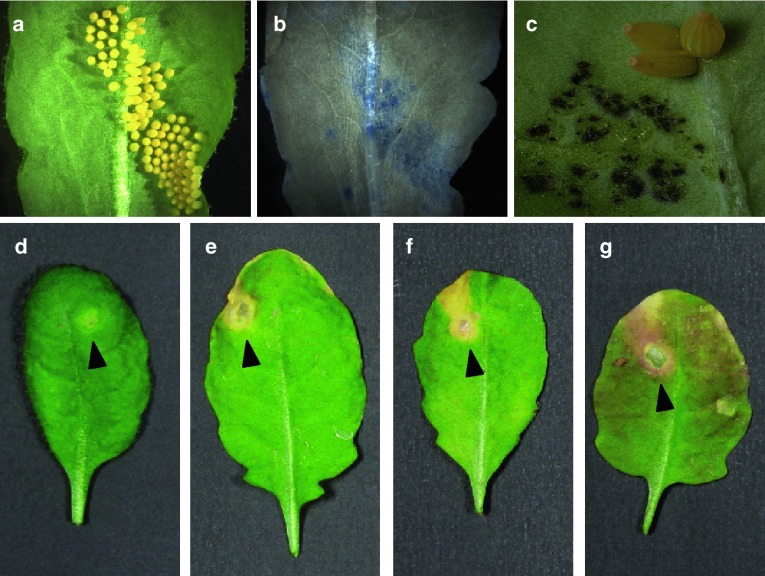
Variability of egg-induced necrosis in *Arabidopsis*. Oviposition by *Pieris brassicae* causes cell death on *Arabidopsis* *thaliana* Col-0 leaves but no strong necrosis can be observed, in contrast to *Brassica oleracea* var *gemmifera*. However, some *Arabidopsis* accessions display a much stronger HR-like necrosis after treatment with *P. brassicae* egg extract. **a** *P. brassicae* eggs on *Arabidopsis thaliana* Col-0; **b** visualization of cell death by trypan blue staining in Col-0 leaves 72 h after oviposition; **c** *B. oleracea* var *gemmifera* leaf 72 h after oviposition, some eggs were removed to show necrosis; **d** Col-0 leaf treated for 72 h with *P. brassicae* egg extract; **e** Ra-0; **f** Bor-4; **g** Lz-0. *Arrows* indicate the site of egg extract deposition

Studies with *Arabidopsis thaliana* showed that oviposition of *P. brassicae* caused a significant cell death underneath the eggs that was detected by trypan blue staining (Little et al. 2007). However, contrary to other members of the Brassicaceae (Hilker and Meiners 2006; Bruessow and Reymond 2007), *P.* *brassicae* eggs did not induce a strong HR-like necrosis in the accession Col-0 but only a yellowish spot underneath the egg mass. To explore the natural variation of this response, a population of *Arabidopsis* accessions was screened and yielded clear differences. Application of crude *P.* *brassicae* egg extract that was shown to mimic intact eggs had almost no visible effects on some accessions, whereas it generated a strong HR-like necrosis in others, which was even spreading beyond the oviposition site (Fig. 2; Gouhier-Darimont et al. unpublished). Generation of recombinant inbred lines between weak (e.g., Col-0) and strong (e.g., Lz-0) responding accessions should help in the future to isolate genetic factors involved in egg-induced HR-like necrosis and to compare them to known components of pathogen-induced HR. It would also be informative to test egg and larval performance in different accessions as well as oviposition responses to *Arabidopsis* mutants impaired in HR, for instance, *dnd1* (Yu et al. 1998), *lsd1* (Dietrich et al. 1997) and *acd2* (Yao and Greenberg 2006).

Finally, pathogen-induced production of ROS is crucial for the establishment of HR (Mur et al. 2008). ROS accumulation has been reported in response to oviposition in several plant species (see above), providing another indication that eggs might be perceived as microbial pathogens, at least at the molecular level.

In summary, a likely scenario is that eggs contain generic elicitors that are recognized like PAMPs and trigger PTI, as described in *Arabidopsis*. Some insect eggs contain additional elicitors that induce highly specific responses in plants that co-evolved with natural enemies (e.g., production of volatiles and modification of leaf surface chemistry). During evolution, some insect species might have generated effectors to interfere with plant defenses and increase their “virulence”. In turn, plants might have evolved resistance genes to target these effectors, leading to ETI. Future research should aim at testing this hypothesis by identifying egg elicitors and effectors and the corresponding plant factors.

## Egg-induced plant defenses and consequences for hatching larvae

Recent studies suggest that plant responses to oviposition are more complex than anticipated, and that they not only have a direct impact on eggs but can also affect defenses against hatching larvae, both positively and negatively. For instance, the emission of oviposition-induced volatiles plays a role beyond the attraction of egg parasitoids. In the African grass *Brachiaria brizantha* and in maize landraces, oviposition by *C.* *partellus* attracted the larval parasitoid *Cotesia sesamiae* (Bruce et al. 2010, 2011; Tamiru et al. 2012). Likewise, oviposition by *P.* *brassicae* on *B. nigra* attracted the larval parasitoid *C.* *glomerata* (Fatouros et al. 2012). The fact that parasitoids are recruited before hatching is quite remarkable and might enhance their chance of finding their host.

In flowering wild crucifer *B. nigra*, oviposition by *P. brassicae* accelerated seed production, even before hatching. Since *P. brassicae* larvae preferably feed on leaves and flowers but not on seeds, this interesting observation is interpreted as a means for the plant to safeguard its reproductive potential (Lucas-Barbosa et al. 2013). In another study looking at the vegetative stage of *B. nigra*, *P. brassicae* eggs were shown to stimulate plant elongation and flowering time, both in laboratory or field conditions (Pashalidou et al. 2013). Again, this response suggests that one way to diminish the negative impact of feeding larvae is to detect eggs early and accelerate seed production. Since this response was not observed with *M.* *brassicae* eggs, whether this strategy is widespread needs to be further investigated.

Since plants and insects have co-evolved for millions of years, a defense response is often counterbalanced by a strategy to avoid or suppress it. There are examples of defense suppression by feeding herbivores (reviewed in Zhu-Salzman et al. 2005) but only few reports identified such phenomenon after oviposition. In *B.* *nigra*, oviposition by the specialist *P.* *brassicae* reduced the emission of the majority of 50 detected plant volatiles, with the exception of a few terpenes, whereas oviposition by the generalist *M.* *brassicae* had almost no effect (Fatouros et al. 2012). The modified volatile profile in response to *P.* *brassicae* oviposition was however correlated with a higher attraction of egg and larval parasitoids compared to uninfested plants (Fatouros et al. 2012). In *B.* *brizantha* exposed to oviposition by *C. partellus*, a significant reduction of (*Z*)-3-hexenyl acetate was the main change in volatile production (Bruce et al. 2010). Again, the consequence of this reduction was apparently not beneficial for the attacking insect since oviposited plants were more attractive to larval parasitoids and less preferred for further oviposition (Bruce et al. 2010). In maize, oviposition by *Spodoptera frugiperda* reduced both constitutive and herbivore-induced volatile terpenoids. Here, the advantage of this suppression by *S.* *frugiperda* was postulated but not assessed (Peñaflor et al. 2011). It is thus unclear whether these suppressions of volatile emission represent an adaptation of the insect to indirect defenses. In these examples, plants might still benefit from the production of other volatile compounds that attract parasitoids and the final outcome of negative and positive effects would be in favor of the plant. Further research will be needed to solve this apparent paradox.

In *Arabidopsis*, treatment with *P. brassicae* or *S. littoralis* egg extracts suppressed the herbivore-induced expression of several defense genes that are controlled by the JA pathway. This effect was due to egg-induced SA accumulation since it was lost in the SA-deficient mutant *sid2*-*1* (Bruessow et al. 2010). The suppression of herbivore-induced genes was also observed with naturally laid *P. brassicae* eggs (F. Bruessow, PhD thesis, unpublished). Remarkably, this suppression was correlated with an enhanced performance of *S. littoralis* larvae, but not *P. brassicae* larvae. Again, this enhanced performance was abolished in *sid2*-*1* (Bruessow et al. 2010). This study demonstrated that insect eggs actively suppress plant defense for the benefit of their own larvae and suggested that eggs hijack the SA pathway to negatively interfere with the JA pathway. The observation that the suppression was ineffective on *P. brassicae* was attributed to the known tolerance of this specialist insect to *Arabidopsis* defenses (Wittstock et al. 2004; Wheat et al. 2007).

On the contrary, oviposition has been shown to protect plants from further attack by hatching larvae. A recent study found that *P. brassicae* larvae that fed on previously oviposited *Arabidopsis* plants consumed less leaf material, gained less weight after 2 days and suffered higher mortality than larvae feeding on plants that did not receive eggs (Geiselhardt et al. 2013). Surprisingly, these results were different from the study published by Bruessow et al. (2010), where *P. brassicae* egg extract pretreatment did not affect the performance of *P. brassicae* larvae after 8 days of feeding. Reasons for this discrepancy might be several. First, Geiselhardt et al. used naturally laid eggs, whereas Bruessow et al. pretreated plants with egg extract. Although it was shown that responses to naturally laid eggs do not differ drastically from egg extract treatment (see above), this might explain the contrasting results. Second, larvae that hatched from egg batches were allowed to feed gregariously on leaves for 2 days, whereas Bruessow et al. placed one larva per plant for 8 days, a less common feeding behavior. Density-dependent priming effects might be activated when several larvae feed on the same leaf. Alternatively, eggs might lower the nutritional quality of a leaf and this might be more detrimental to a group of larvae compared to a single one that would have a better access to appropriate nutrients. Molecular or chemical changes that could explain the enhanced resistance of naturally oviposited *Arabidopsis* plants to *P.* *brassicae* larvae were however not identified and further studies should explore this question. On the contrary, levels of a major anti-insect defense metabolite, the 4-methylsulfinylbutyl glucosinolate, and the expression of some glucosinolate biosynthesis genes were significantly reduced in damaged leaves with prior oviposition (Geiselhardt et al. 2013), in line with the suppression of herbivore-induced genes found by Bruessow et al. (2010). It would be interesting to test if natural oviposition by generalist herbivores that lay eggs on *Arabidopsis* triggers a similar response and whether a reduced glucosinolate content enhances larval performance, as would be expected from the known role of these metabolites.

Other examples of egg-induced plant protection are known. When *D.* *pini* sawfly larvae were feeding on pine twigs from which they hatched, they gained significantly less weight and had increased mortality compared to feeding on egg-free twigs. In addition, adult fecundity was reduced (Beyaert et al. 2012). Although *TPS1* and *TPS2* expression peaked just before hatching, this was not correlated with significant changes in terpenoid and phenolic metabolites. Thus, a protective effect of oviposition could not be assigned to known defense metabolites and the mechanism of this interesting observation deserves further investigation. In tomato, oviposition by the fruitworm *H. zea* primed the wound-induced expression of *PIN2* and JA accumulation, which are typically involved in resistance against feeding herbivores (Kim et al. 2012). Since *H.* *zea* does not damage plants during egg deposition, this phenomenon was attributed to the presence of egg-associated factors. Given that H_2_O_2_ accumulated underneath the eggs and that H_2_O_2_ activates the JA pathway in tomato (Orozco-Cárdenas et al. 2001), the authors postulated that ROS production might be responsible for this effect (Kim et al. 2012). They, however, did not evaluate insects’ performance after oviposition and thus the relevance of these findings has yet to be demonstrated. Growth of specialist *P. brassicae* and generalist *M. brassicae* larvae was significantly reduced when feeding on *B.* *nigra* leaves that received *P.* *brassicae* eggs compared to non-oviposited leaves (Pashalidou et al. 2013). This induced resistance was observed both in the laboratory and in a common garden field plot. In contrast, oviposition by *M.* *brassicae* had no effect on further performance of *M.* *brassicae* or *P.* *brassicae* larvae. Although these data provide clear evidence that eggs from a specialist herbivore can protect *B. nigra* plants from further herbivory, molecular events underlying this process were not assessed. It would be interesting to see if JA accumulation and defense gene expression are primed by oviposition and if it only occurs with adapted insects.

Besides triggering direct and indirect defenses, there is thus accumulating evidence that insect eggs can either manipulate plant signaling pathways for their own benefit or prepare a plant for further feeding damage. This is another illustration of the on-going arms race that governs interactions between plants and insects, where in some instances the herbivore can overcome a specific plant defense and warrant a good start in life for neonate larvae, while on the other hand plants can anticipate an attack by responding to an inert stage of their enemy.

## Conclusions and suggestions for future research

**Fig. 3:**
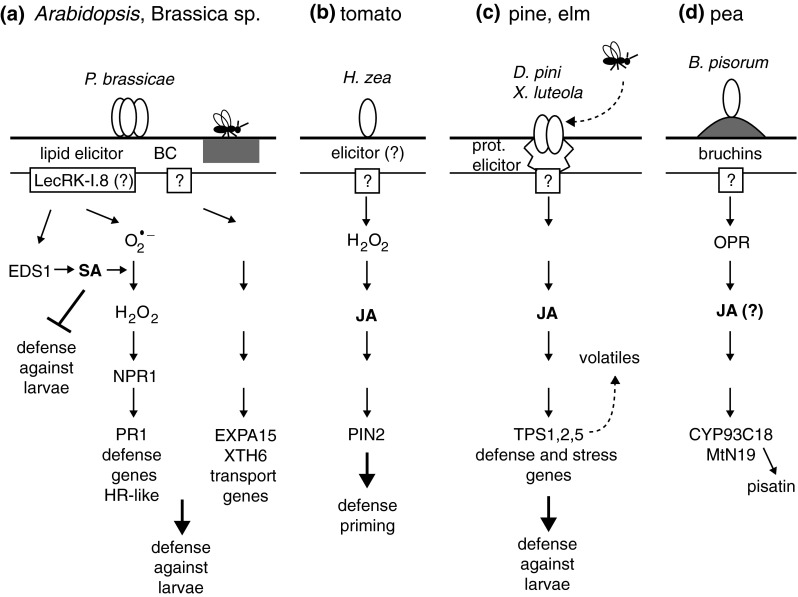
Summary of the current knowledge on egg perception, signaling and defense gene expression. Typical responses of plants to oviposition are shown. **a** In plants from the Brassicaceae family, eggs deposited on the leaf surface release elicitors that are either contained in the egg (lipid elicitor) or found in secretions coating the eggs (benzyl cyanide, BC). In *Arabidopsis*, after binding to the putative LecRK-I.8 receptor, a lipid elicitor triggers the SA pathway that in combination with ROS activates the expression of defense genes, including *PR1*, and an HR-like necrosis. Egg-induced SA accumulation suppresses JA-dependent defenses against larvae from a generalist herbivore. In addition, BC triggers the expression of cell wall metabolism and transport genes that results in leaf surface chemical changes (*gray*) arresting egg parasitoids. In *Brassica nigra* and *Arabidopsis*, oviposition leads to induced resistance against larvae from a specialist herbivore. **b** In tomato, oviposition triggers H_2_O_2_ accumulation and expression of the JA-dependent *PIN2*. Eggs prime plants for enhanced JA accumulation and defense gene expression in response to further herbivory. **c** In pine and elm trees, a combination of wounding and proteinaceous elicitors present in secretions coating the eggs induce the emission of plant volatiles and the activation of stress and defense genes through the JA pathway. These terpenoids are synthesized by terpene synthase genes (TPS) and attract egg parasitoids. In addition, oviposition by pine sawfly *Diprion pini* decreases further larval performance and adult fecundity. **d** In pea, bruchins found in oviposition fluids of the bruchid weevil induce neoplasm formation (*gray*), activate gene expression and stimulate the accumulation of the defense compound pisatin. The induction of an OPDA-reductase gene (OPR) suggests that the JA pathway is involved in these responses

Over recent years, the field of plant responses to oviposition has seen significant progress in the understanding of egg perception, downstream signaling steps and transcriptional changes that control direct and indirect defenses. From a passive substrate, plants have become highly sensitive and sophisticated organisms that actively recognize and defend against an early stage of insect attack. Plant–egg interactions show attributes of an innate immune response, including the detection of generic egg-associated molecular patterns, PTI signaling and HR-like necrosis. In addition, species-specific responses involving tritrophic interactions imply another level of complexity requiring specific elicitors. Figure 3 presents a summary of the current knowledge of egg perception, signal transduction and defense gene expression. Yet, many questions remain unanswered:

- What is the chemical nature of elicitors contained in eggs or associated with coating secretions?
- Which plant receptors detect egg-derived elicitors?
- Do eggs deliver effectors to suppress plant defenses?
- What is the specific contribution of SA and JA pathways downstream of egg perception?
- Which genes are involved in direct and indirect defenses?
- What is the genetic basis of HR-like necrosis development?
- What are the molecular mechanisms of egg-induced resistance against feeding larvae?
- How is specificity of certain plant–egg interaction achieved?
- What is the outcome of oviposition by generalist or specialist insects?

The increasing availability of genomic tools has the potential to help answering these questions. *Arabidopsis* has proven useful to analyze responses that are triggered by compounds from eggs deposited on the leaf surface. Unfortunately, there is no study on egg-induced volatiles in *Arabidopsis*, although it was shown that herbivory triggers the emission of terpenoids, methyl salicylate and green leaf volatiles (Van Poecke et al. 2001; Snoeren et al. 2010). With its large collection of mutants and natural accessions, this species could represent an interesting model to analyze the involvement of egg-induced volatiles in defense. However, other model species will be needed to explore other types of oviposition. Finally, there is a need to bridge molecular approaches and ecological studies to get more insights into this fascinating interaction.

## Abbreviations

ARG: Accessory reproductive gland
BC: Benzyl cyanide
ET: Ethylene
ETI: Effector-triggered immunity
HR-like: Hypersentive response-like
JA: Jasmonic acid
PAMP: Pattern-associated molecular pattern
PTI: Pattern triggered immunity
ROS: Reactive oxygen species
SA: Salicylic acid
